# Variability of noninvasive MRI and biological markers in compensated cirrhosis: insights for assessing disease progression

**DOI:** 10.1186/s41747-022-00303-y

**Published:** 2022-10-24

**Authors:** Christopher R. Bradley, Eleanor F. Cox, Naaventhan Palaniyappan, Guruprasad P. Aithal, Susan T. Francis, Indra Neil Guha

**Affiliations:** 1 NIHR Nottingham Biomedical Research Centre, Nottingham University Hospitals NHS Trust and the University of Nottingham, Nottingham, UK; 2 Sir Peter Mansfield Imaging Centre, School of Physics and Astronomy, University of Nottingham, Nottingham, UK; 3 Nottingham Digestive Diseases Centre, School of Medicine, University of Nottingham, Nottingham, UK

**Keywords:** Biomarkers, Disease progression, Liver cirrhosis, Multiparametric magnetic resonance imaging, Sample size

## Abstract

**Background:**

We annually monitored stable compensated cirrhosis (CC) patients to evaluate serial variation in blood serum, liver stiffness, and multiparametric magnetic resonance imaging (mpMRI) measures to provide reference change values (RCV) and sample size measures for future studies.

**Methods:**

Patients were recruited from a prospectively followed CC cohort, with assessments at baseline and annually over three years. We report on blood markers, transient elastography liver stiffness measures (LSM) and noninvasive mpMRI (volume, T1 mapping, blood flow, perfusion) of the liver, spleen, kidneys, and heart in a stable CC group and a healthy volunteer (HV) group. Coefficient of variation over time (CoV_T_) and RCV are reported, along with hazard ratio to assess disease progression. Sample size estimates to power future trials of cirrhosis regression on mpMRI are presented.

**Results:**

Of 60 CC patients enrolled, 28 with stable CC were followed longitudinally and compared to 10 HVs. CoV_T_ in mpMRI measures was comparable between CC and HV groups. CoV_T_ of Enhanced Liver Fibrosis score was low (< 5%) compared to Fibrosis-4 index (17.9%) and Aspartate Aminotransferase-to-Platelet-Ratio Index (19.4%). A large CoV_T_ (20.7%) and RCV (48.3%) were observed for LSM. CoV_T_ and RCV were low for liver, spleen, and renal T1 values (CoV_T_ < 5%, RCV < 8%) and volume (CoV_T_ < 10%, RCV < 16%); haemodynamic measures were high (CoV_T_ 12–25%, RCV 16–47%).

**Conclusions:**

Evidence of low CoV_T_ and RCV in multiorgan T1 values. RCV and sample size estimates are provided for future longitudinal multiorgan monitoring in CC patients.

**Trial registration:**

ClinicalTrials.gov identifier: NCT02037867, Registered: 05/01/2013.

## Key points

- Liver, kidney, and spleen T1 have low variation over time in stable compensated cirrhosis (CC).
- Multiorgan haemodynamic multiparametric magnetic resonance imaging (mpMRI) measures have high variation over time in stable CC.
- Liver T1, volume, blood flow, and spleen volume were predicted to best detect cirrhosis progression.
- Liver T1 and left ventricle wall mass were predicted to best detect decompensation progression.
- Sample size estimates for future multiorgan mpMRI trials of CC regression are provided.

## Background

The assessment of chronic liver disease using noninvasive markers is firmly established within clinical practice to study liver fibrosis across aetiology [1–4]. Baseline measures of chronic liver disease have been shown to provide prognostic value in determining clinical outcomes [5], using simple laboratory tests [6, 7], specific fibrosis markers [8], and transient elastography (Fibroscan®) [6, 9]. Recently, novel magnetic resonance imaging (MRI) techniques [10] have been used to study liver disease, for example liver tissue longitudinal relaxation time, *i.e.*, T1, has been shown to correlate with disease severity of liver fibrosis in a cross-sectional study [11]. Liver MRI is now being used in clinical trials of longitudinal change, and the need to study critical organs such as the heart, kidneys and splanchnic circulation is now recognised as a central aspect in the clinical management of cirrhotic patients [12–14]. However, there is limited knowledge of serial variation of multiorgan MRI measures in healthy volunteers and stable patients. It is important to know whether the increase or decrease between two measurements collected serially in time is of clinical significance.

The serial change in a measurement originates from its technical variation (related to the test imprecision) and the biological variation within a subject over time - together this is described by the intra-individual coefficient of variance across time (CoV_T_), as well as any change in pathology due to disease progression or regression. For serial measures to reflect clinical improvement or disease progression, any changes over time in the absolute value of a measure should exceed the CoV_T_.

The measurement of biomarkers in stable liver disease provides an insight into the temporal variation in the serial, longitudinal measurement of a biomarker, and the required change needed to reflect an alteration in the underlying pathology. Studies reporting the variation in measures in stable patients with liver disease are limited exclusively to blood tests and serum markers [15–17]. In a recent paper, Trivedi et al. [18] assessed the inter- and intra-individual variation in serum alkaline phosphatase and enhanced liver fibrosis (ELF) score over time in patients with primary sclerosing cholangitis. They showed that serum alkaline phosphatase could not associate with disease progression due to a large CoV_T_, whilst the ELF score had a lower CoV_T_ of < 5% and could be used to track fibrosis progression and development of cirrhosis. To date, very few studies have reported the variation of MRI measures in chronic liver disease [19, 20].

The aim of this study is to assess the serial annual variation in blood serum and multiorgan MRI measures of blood flow, perfusion, volume, and T1 mapping in the liver, kidney, and spleen in a stable compensated cirrhosis (CC) patient group who did not develop any clinical outcomes, *i.e.*, in the absence of decompensation and stable validated measures of liver function: model for end-stage liver disease (MELD) and United Kingdom model for end-stage liver disease (UKELD). These data provide reference change values in compensated cirrhosis patients to track disease progression or regression and for intervention assessment in future studies.

## Methods

### Study design and cohort information

We performed a retrospective analysis of data from individuals enrolled in the Compensated Cirrhosis Cohort in Nottingham (3CN Study), a study focused on tracking liver disease [10] (research approval 10/H0403/10). Inclusion criteria were evidence of cirrhosis based on histology and radiological features and no evidence of decompensation (ascites, significant jaundice, hepatic encephalopathy, and variceal bleeding), hepatocellular carcinoma, and portal vein thrombosis. Exclusion criteria included orthotopic liver transplantation, ischaemic heart disease, alcoholic cardiomyopathy (defined by clinical evidence of systolic dysfunction) and valvular heart disease (defined by echocardiography). CC patients were managed in accordance with standard clinical care guidelines [21]. For alcoholic and non-alcoholic fatty liver disease (NAFLD), lifestyle intervention was offered including referral to an alcohol counsellor and/or dietician, both with a special interest in chronic liver disease. For patients with chronic hepatitis C, the treatment regimens followed the national guidelines appropriate to the specific area (directly acting anti-viral treatment). In addition, patients attended six-monthly clinical research visits (physical examination, blood tests, and Fibroscan®) and an annual research multiparametric MRI (mpMRI).

**Fig. 1:**
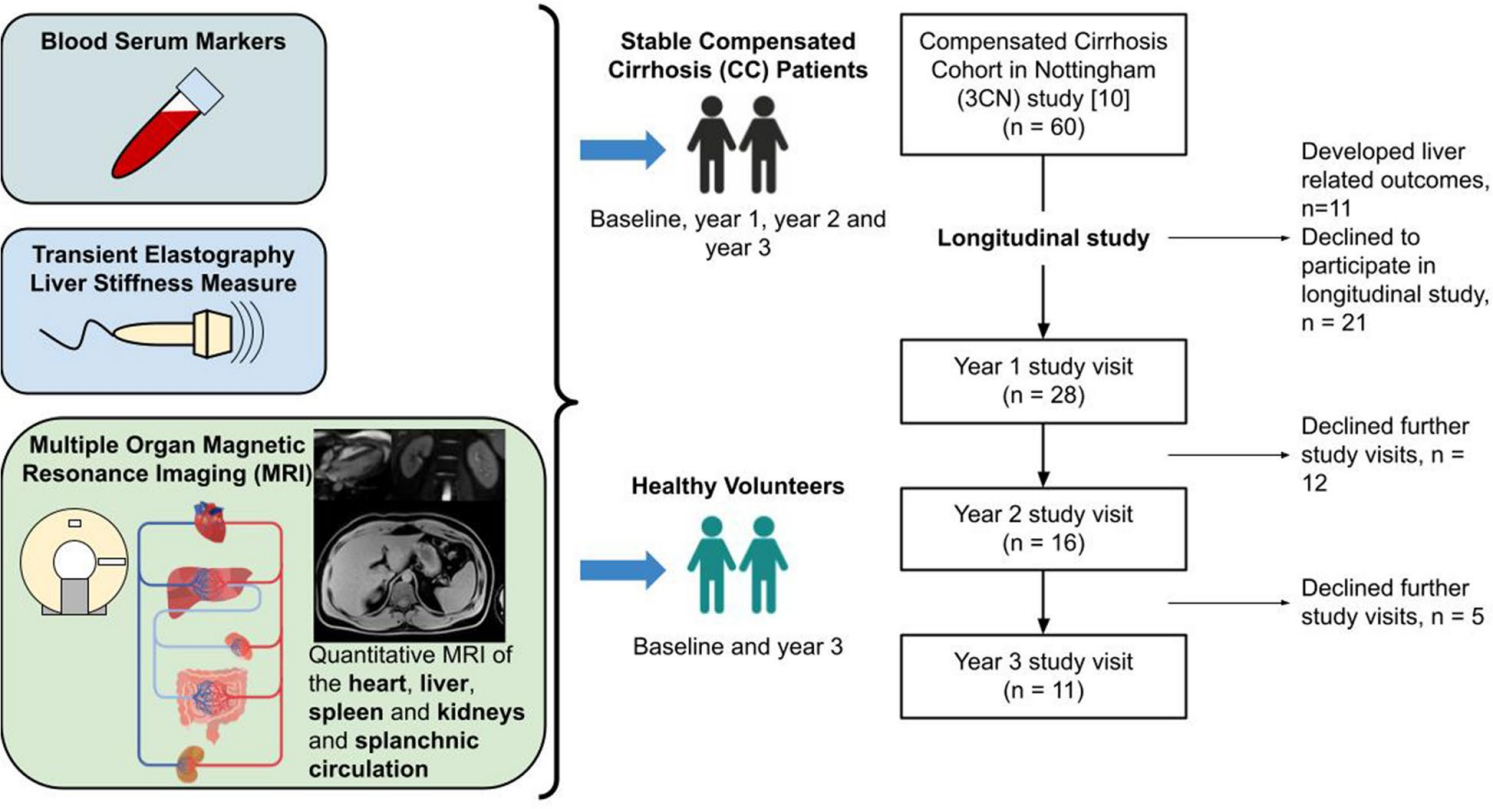
Schematic of the study and consort diagram. Schematic of blood markers (MELD, UKELD, APRI, FIB4, ELF), Fibroscan® LSM, and multiparametric magnetic resonance imaging (volume, T1-mapping, blood flow, perfusion) of the liver, spleen and kidneys, and cardiac index. Illustration of healthy volunteers (HV) and compensated cirrhosis (CC) patients studied longitudinally as indicated by consort diagram. *MELD* Model for end-stage liver disease, *UKELD* United Kingdom model for end-stage liver disease, *APRI* Aspartate aminotransferase to platelet ratio index, *FIB4* Fibrosis-4, *ELF* Enhanced liver fibrosis, *LSM* Liver stiffness measure, *TE* Transient elastography

Patients were assessed at baseline and returned for research visits for up to 3 years. Of the 60 CC patients scanned at baseline, 28 formed our stable control CC group. They did not develop any clinical outcomes (absence of decompensation and stable validated MELD or UKELD measures of liver function) and accepted to take part in longitudinal follow-up are studied in this work. Figure 1 provides an overview of the study protocol and annual research visits, illustrating 28 control CC patients at year 1, with 11 completing their year 3 follow-up. In addition, of the 40 healthy volunteers (HVs) assessed at baseline, 10 HVs who were age, gender, and body mass index (BMI) matched to the year 3 CC patient group were scanned at baseline and year 3. Decompensated cirrhosis (DC) patients were scanned at baseline only.

At each visit, participants attended following an overnight fast. Blood samples assessed markers of liver fibrosis including Enhanced Liver Fibrosis (ELF), Aspartate aminotransferase to Platelet Ratio Index (APRI) and Fibrosis-4 (FIB4) index. mpMRI measures detailed below were collected. In addition, Fibroscan® liver stiffness measure (LSM) was obtained by an experienced operator. For the HV group, only ELF and MRI measures were collected.

### Multiorgan mpMRI protocol

Participants were scanned following a 6-hour fast, between 8 am and 12 pm. All imaging was performed on a 1.5-T Achieva scanner (Philips, Best, The Netherlands) using a 16-element Torso receive coil and the body transmit coil. In each 1-hour scan session, unenhnaced MRI measures were collected on the liver (~20 min), splanchnic organs, and kidneys (~15 min), and heart (~10 min) [10]. Imaging sequence parameters for all MRI measures are shown in detail in Table 1. This comprised: liver-portal vein and hepatic artery blood flow, liver perfusion, and tissue T1 [11, 22]; spleen and superior mesenteric artery–splenic artery and superior mesenteric artery blood flow, splenic tissue perfusion and tissue T1; kidney–right renal artery blood flow, kidney volume, renal tissue perfusion [23, 24], and tissue T1 [24]; heart–cardiac index and left ventricular (LV) wall mass index [25]. Organ volume was measured from high resolution anatomical images. T1 mapping was performed using a respiratory triggered inversion recovery spin echo echo-planar imaging scheme. Blood flow measures were performed using phase-contrast MRI and perfusion using respiratory triggered flow alternating inversion recovery arterial spin labelling (ASL). Due to the longitudinal repeat measures performed in this study, no dynamic contrast-enhanced MRI measures were collected.

**Table 1:** Imaging sequence parameters for all magnetic resonance imaging (MRI) measures used in this study

Parameter	Sequence	Readout	Echo time/TR (ms)	FoV (mm^3^)	Voxel size (mm^3^)		Sequence specific	Scan duration (s)
**Volume**						Orientation		
Liver, spleen, kidneys	Anatomical	BTFE	1.5/3.3	400 × 400 × 175	1.56 × 1.56 × 7	Coronal		18
**Blood flow**						VENC (cm/s)	Phases	
Superior mesenteric artery	PC-MRI	TFE	3.7/6.9	150 × 280 × 6	1.17 × 1.17 × 6	140	15	15−20
Hepatic artery	PC-MRI	TFE	3.7/6.9	150 × 280 × 6	1.17 × 1.17 × 6	100	15	15−20
Splenic artery	PC-MRI	TFE	3.7/6.9	150 × 280 × 6	1.17 × 1.17 × 6	100	15	15−20
Right renal artery	PC-MRI	TFE	3.7/0.9	150 × 280 × 6	1.17 × 1.17 × 6	100	15	15−20
Portal vein	PC-MRI	TFE	3.7/6.9	150 × 280 × 6	1.17 × 1.17 × 6	50	15	15−20
**Cardiac**						VENC (cm/s)	Phases	
Cardiac Output	PC-MRI of ascending aorta	TFE	3.7/6.9	280 × 280 × 6	1.17 × 1.17 × 6	200	30	60
Left ventricle wall mass	Short-axis cine MRI	TFE	1.25/2.5	370 × 370 × 96	2 × 2 × 8	Oblique	30	4′ 15−20 s
**Organ Perfusion**							ASL parameters	
Liver	FAIR-ASL	BFFE	1.2/2.4	288 × 288 × 39	3 × 3 × 8	Sagittal right lobe	TI 1,100 ms, 60 pairs	~ 360
Spleen and Kidneys	FAIR-ASL	BFFE	1.2/2.4	288 × 288 × 25	3 × 3 × 5	Coronal oblique through kidney long axis	TI 1,100 ms, 30 pairs	~ 180
**Microstructure**							Inversion times (ms)	
Liver	Inversion recovery T1 mapping	FS SE-EPI	27/8,000	288 × 288 × 39	3 × 3 × 8	Sagittal right lobe	100–1,200 ms in 100 ms steps + 1,500 ms	~120
Spleen and kidneys	Inversion recovery T1 mapping	BFFE	1.2/2.4	288 × 288 × 25	3 × 3 × 5	Coronal oblique through kidney long axis	100–900 ms in 100 ms steps	~120

*BFFE* Balanced fast field-echo, *BTFE* Balanced turbo-field-echo, *FAIR ASL* Flow alternating inversion-recovery arterial spin labelling, *FoV* Field of view, *FS* Fat-suppressed, *PC-MRI* Phase-contrast MRI, *SE-EPI* Spin-echo echo-planar imaging, *TFE* Turbo field-echo, *TI* Inversion time, *TR* Repetition time, *VENC* Velocity encoding

### Image analysis

#### Volume of liver, spleen and kidneys

Analyze® (Version 9, Mayo Clinic https://analyzedirect.com/) was used to draw an ROI around each organ (liver, kidney, spleen) for each slice, with total organ volume calculated by summing across slices. Liver and spleen volumes were adjusted for patient body surface area (BSA).

#### Blood flow measures

MR Qflow (a plug-in available on the ViewForum Philips Medical Systems, Best, The Netherlands https://www.philips.co.uk/healthcare/product/HCAPP013/-mr-qflow-) was used to analyse phase-contrast MRI data. For each vessel, a region of interest was drawn on each cardiac phase to estimate flow by averaging the flow velocity values and multiplying by vessel lumen cross-sectional area. Mean flow was calculated by averaging flow across cardiac cycle phases.

#### Cardiac function and structure

Cardiac MRI data was analysed using ViewForum software (Philips Medical Systems, Best, The Netherlands). phase-contrast MRI data of the aorta was analysed by computing stroke volume and heart rate, then multiplying these to yield cardiac output. This software was also used to draw wall contours to calculate end diastolic LV wall mass. Cardiac output and LV wall mass were adjusted for patient BSA[NO_PRINTED_FORM].

#### Perfusion and T1 relaxation mapping of the liver, spleen and kidneys

Inversion-recovery data were fit to a two-parameter model to generate T1 maps. ASL analysis was performed using MATLAB (2014a, Natick, MA, USA) and/or IDL (version 8.0, Broomfield, CO, USA). Individual perfusion-weighted images (control-label) were calculated, inspected for motion (excluding > 1 voxel) and averaged creating a perfusion-weighted image. Perfusion-weighted image, base equilibrium magnetisation, M_0_, image, and T1 maps were used in a kinetic model [26] to compute tissue perfusion maps.

A binary organ mask was formed to calculate mean liver, spleen and renal cortex perfusion. For the liver and spleen, masks were formed from the base M_0_ image and applied to T_1_ maps to obtain the median T1 (excluding major blood vessels with a T_1_ > 1,300 ms) and perfusion. Whole kidney masks were formed by manual segmentation of the T1 map, applied to the T1 and perfusion maps, and the mode of each parameter calculated for each kidney, the mean was then computed across kidneys.

### Statistical analysis

In this stable CC control group, we aim to determine the variance over time in blood serum, Fibroscan® LSM and MRI measures of blood flow, structure, and perfusion, and evaluate this in the context of future clinical trials. To address this, we performed the following analyses regarding (1) the changes with disease stage in the baseline cross-sectional data; (2) the coefficient of variation (CoV) within the stable CC control group at baseline (interindividual, groupwise CoV, CoV_G_); (3) the CoV across time in annual measurements in individuals in the stable CC control group (intraindividual, CoV_T_) and the reference change value (RCV), defined as the percentage change in a measure in an individual that can be attributed to pathological change, (as employed in a recent study of high sensitivity cardiac troponin-T (hs-cTnT) concentration in dialysis patients [27]); (4) the variation in serum and MRI measures collected in HVs to determine whether the results differ between stable CC and HV group; (5) the hazard ratio (HR) of these measures to assess disease progression; (6) sample size estimates for future longer-term clinical trials powered on mpMRI measures to study cirrhosis regression. Details of each of these analyses are provided below.

1. *Cross-sectional multiparametric MRI measures with disease stage.* The percentage change in MRI measures at baseline between the HV, CC, and DC groups was calculated to determine clinically relevant increases or decreases in measures.
2. *Categorical* *change in clinical and MRI measures in the stable CC control group.* To demonstrate the stability of each clinical and MRI measure in the stable CC control group, the percentage change from baseline was computed for each individual at year 1, year 2, and year 3. After a Shapiro-Wilk test for normality, a paired Wilcoxon test confirmed no significant changes between time-points (Prism 8, GraphPad Software, Inc., La Jolla, CA, USA). The median (interquartile range, IQR) group percentage change from baseline at year 1, year 2, and year 3 was then calculated. For each measure, the CoV within the group at baseline was computed (inter-individual, CoV_G_).
3. *Annual* *intra-individual variability in clinical and MRI measures in the stable CC control group.* The year-to-year intra-individual variation in each measure was assessed by calculating the annual CoV in each of the measures (year 1 *versus* baseline, year 2 *versus* year 1, year 3 *versus* year 2), and computing the median of these CoVs defined to be the CoV_T_ (intraindividual variance across time). Importantly, this variation combines the effect of the intraindividual biological variation and analytical sample measurement error (from two repeat measurement collected sequentially in time). The RCV was then computed as follows:
  $$\mathrm{RCV}={2}^1/2\times \mathrm{Z}\times {\mathrm{CoV}}_{\mathrm{T}}$$

For a significant (*p* < 0.05) one-directional change a *Z*-score (*Z*) of 1.65 was used, and the log-normal approach was used to compute the asymmetrical limits for the upward (positive, RCV_up_) and downward (negative, RCV_down_) value of the log-normal RCV.

4. *Baseline to Year 3 intra-individual variability in clinical and MRI measures in the stable CC control group and HV group.* To assess whether the timing and frequency of sampling are important factors in CoV_T_ and RCV measures, CoV_T_ was also computed for the stable CC control group from the baseline and Year 3 measures only, this was also performed for the HV group. The median intra-individual CoV across each group was then computed for CoV_T_. For each measure, RCV and the asymmetrical limits were computed from CoV_T_. The coefficient of variance at baseline (CoV_G_) was also calculated for each group.
5. *Performance of measures to detect disease progression using MRI.* To evaluate the sensitivity to assess progression from HV to CC, a HR was computed for each MRI measure, defined as the difference in absolute values of a measure between HV and CC groups divided by the RCV in absolute units for that measure in the HV group. We also compute the HR for progression from CC to DC using the absolute value of the RCV of the CC group. A positive HR indicates an increase in the absolute value of a measure, whilst a negative HR indicates a decrease. A HR > 1 or < -1 suggests that the MRI measure could detect a significant pathological change.
6. *Sample size estimation for clinical trials detecting regression of cirrhosis using MRI.* To illustrate how MRI measures could be used in clinical trials, the sample size needed to detect a clinically significant change from compensated cirrhosis (F4) to advanced fibrosis (F3) at a power of 80% and confidence of 0.05 was calculated. For this, we extrapolate from the change in T1 from F4 to F3 reported in our previous work with biopsy-proven measures [11]. This showed a change of 55 ms for T1 from F4 to F3, equivalent to 50% of the change in T1 from CC toward HVs reported in this study. Thus, to represent F4 to F3 in other measures we also report the sample size needed for a 50% change from CC toward HV.

## Results

### Cross-sectional multiorgan mpMRI measures with disease stage

**Fig. 2:**
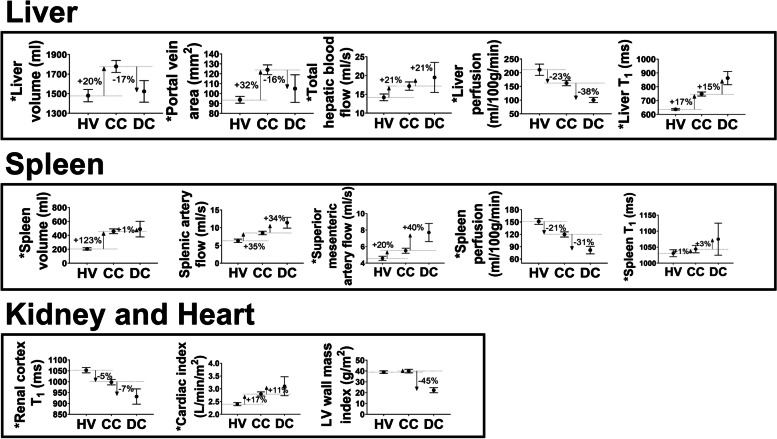
Baseline magnetic resonance imaging parameters for 40 healthy volunteers (HV), 60 compensated cirrhosis (CC) patients, and 7 decompensated cirrhosis (DC) patients. Baseline measures (mean and standard error of the mean) of the liver (volume, portal vein area, total hepatic blood flow, liver perfusion, liver T1), spleen (volume, splenic artery flow and superior mesenteric artery flow, spleen perfusion, spleen T1), kidney (renal cortex T1), and heart (cardiac index and left ventricle [LV] wall mass index) are shown, with the percentage change between the HV and CC groups, and CC and DC groups shown by arrows. Asterisk indicates measures which are significantly different (*p* < 0.05, independent samples *t*-test) between the CC and HV group [10]

At baseline, cross-sectional multiorgan MRI measures were collected in 60 CC patients and 40 HVs, and DC patients. In patients with CC, a hyperdynamic circulation resulted in increased blood flow in the liver, splanchnic circulation and increased cardiac index, with further increases in spleen blood flow and cardiac index in patients with DC, as summarised in Fig. 2. Liver and splenic perfusion was reduced in patients with CC compared to the HV group, and perfusion in these organs was further reduced in those with DC. No significant change in renal perfusion was found between patients with CC and DC, and the HV group. Liver tissue T1 increased in patients with CC compared to HVs, and further increased in those with DC. Spleen T1 was only significantly different from the HV group in DC patients. In contrast, renal T1 reduced in patients with CC and further reduced in those with DC, compared to HVs. LV wall mass was significantly reduced in patients with DC compared to HVs, whilst liver volume was found to increase only in patients with CC, and spleen volume was increased in patients with CC and DC compared to HVs. Note that with disease progression liver volume and portal vein area first increase from HV to CC and then decrease as patients decompensate. This baseline cross-sectional data has previously been described in detail [10]. These results provide the context for the percentage change in MRI measures between the HV, CC and DC groups, and the increase or decrease in measures that are of clinical relevance when considering longitudinal variance of measures.

### Baseline characteristics of the stable CC control group and HV group

Of the 60 CC patients enrolled at baseline, on retrospective analysis, 28 stable CC control patients were followed longitudinally. The baseline characteristics of this 28 stable CC group along with the 32 non-returners are provided in Table 2. The stable CC control group comprised 28 patients at year 1, 16 patients at year 2 and 11 patients at year 3. Of the 28 stable CC control patients, 21 patients (75%) had cirrhosis diagnosed from liver biopsy, 4 (14%) from typical radiological features of cirrhosis (nodular liver and evidence of portal hypertension), and 3 (11%) from clinical findings of cirrhosis including typical clinical history and presence of abdominal collaterals on examination.

**Table 2:** Characteristics of the 60 compensated cirrhosis cohort divided into 28 stable control patients followed longitudinally in this paper, and those non-returner patients excluded as they either failed to return for repeat assessments or developed a clinical outcome. Also shown is the 10 healthy volunteer group followed longitudinally

	Compensated cirrhosis		Healthy volunteers
	Stable returners	Non-returners	
N	28	32	10
Gender	17 male (61%)	18 male (56%)	6 male (60%)
Age (years)	59 (8)	59 (13)	63 (4)
Aetiology	29% NALFD/18% ALD/25% HCV/28% other	40% NAFLD/37% ALD/15% HCV/7% other	N/A
BMI (kg/m^2^)	27.5 (5.6)	28.5 (4.9)	26 (3.0)
MELD	7.5 (1.6)	6.9 (1.8)	N/A
UKELD	43.1 (2.9)	43.5 (2.3)	N/A
APRI	0.71 (0.61)	0.71 (1.2)	N/A
FIB4	2.5 (1.8)	2.9 (1.9)	N/A
ELF score	10.2 (1.2)	11.5 (1.9)	8.9 (0.8)
Fibroscan® LSM (kPa)	18 (17)	23 (21)	N/A

Numbers in parentheses correspond to the standard deviation in measures unless stated otherwise

*NAFLD* Non-alcoholic fatty liver disease, *ALD* Alcoholic liver disease, *HCV* Hepatitis C virus, *BMI* Body mass index, *MELD* Model for end-stage liver disease, *UKELD* United Kingdom model for end-stage liver disease, *APRI* Aspartate aminotransferase to platelet ratio index, *FIB4* Fibrosis-4, *ELF* Enhanced liver fibrosis, *LSM* Liver stiffness measure

There was no significant change in the BMI during the study period, with a median BMI of 28 (IQR 6), 28 (IQR 6), and 27 (IQR 4) kg/m^2^ at years 1, 2, and 3 respectively. All patients with chronic hepatitis C achieved sustained virological response prior to the study. In alcohol-related cirrhosis, abstinence from alcohol was noted among all the patients. In addition, 10 age, gender, and BMI-matched healthy volunteers were studied at baseline and year 3, whose baseline characteristics are provided in Table 2.

### Annual intra-individual variability in clinical measures in the stable CC control group

**Fig. 3:**
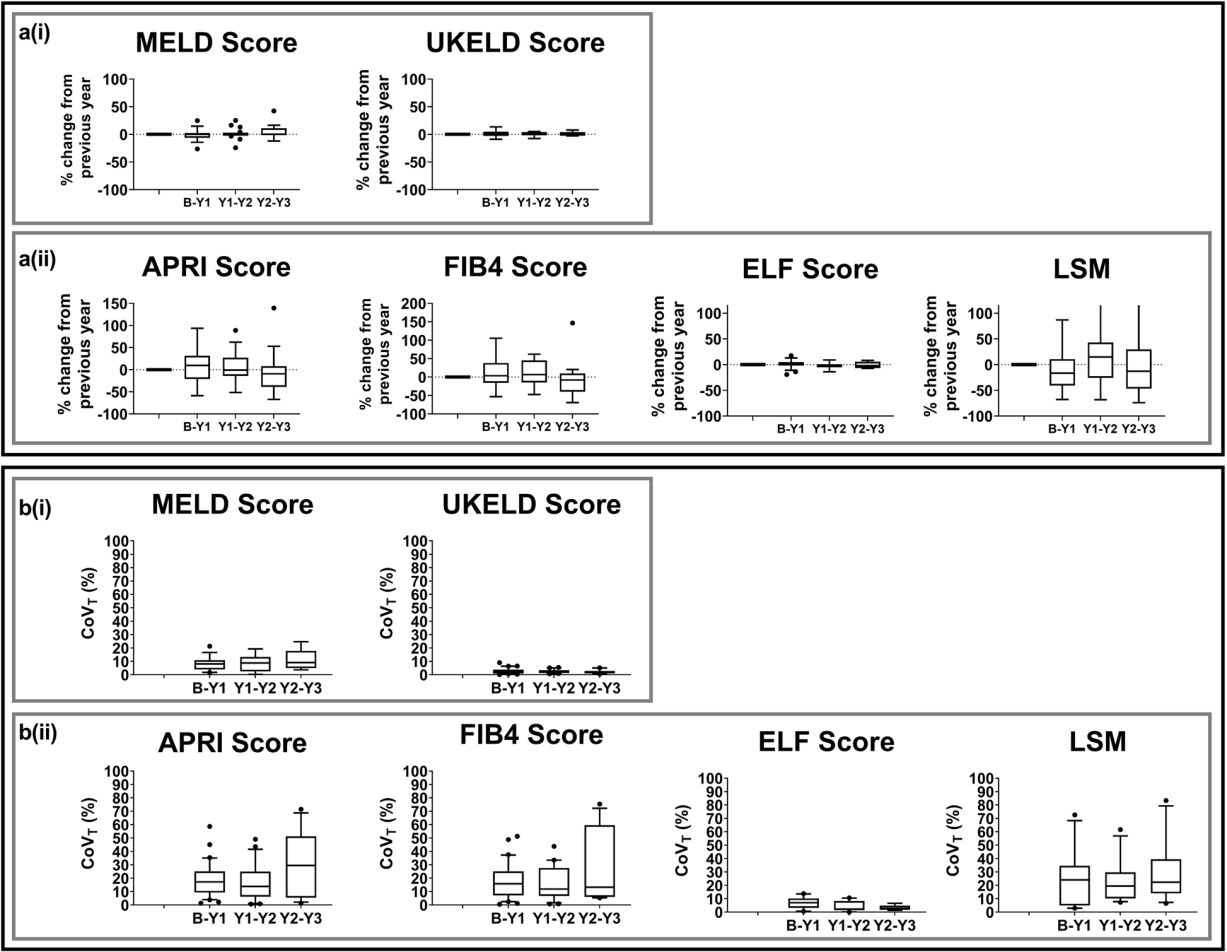
**a** Year-to-year percentage change in clinical measures in the stable compensated cirrhosis control group. **b** Year-to-year coefficient of variation (CoV_T_) in clinical measures in the stable compensated cirrhosis control group. Clinical measures of MELD, UKELD, APRI, FIB4, ELF scores, and Fibroscan® LSM are shown. Bars indicate the interquartile range and the horizontal bold line shows the median, dots represent outliers. *MELD* Model for end-stage liver disease, *UKELD* United Kingdom model for end-stage liver disease, *APRI* Aspartate aminotransferase to platelet ratio index, *FIB4* Fibrosis-4, *ELF* Enhanced liver fibrosis, *LSM* Liver stiffness measure

Figure 3a shows the year-to-year percentage change in MELD and UKELD scores, APRI, FIB4, ELF, and Fibroscan® LSM. There were no significant (*p* ≥ 0.23) changes in MELD, UKELD, APRI, FIB4, ELF score or Fibroscan® LSM over the three years. Figure 3b shows the year-to-year CoV_T_ and Table 3 summarises the CoV_G_, CoV_T_, and RCV in the clinical measures in the stable CC control group. For all clinical measures, CoV_T_ was lower than CoV_G_. UKELD and ELF score showed the lowest annual variation with a median CoV_T_ of 2.2% and 4.0% respectively. Fibroscan® LSM had the largest annual variation with median CoV_T_ of 20.7%. The RCV values provide the percentage change in a serial measurement that represents a statistically significant (*p* < 0.05) change. The RCV for UKELD and ELF score was 5.1% and 6.8%, respectively, whilst the RCV for Fibroscan® LSM was markedly higher at 48.3%.

**Table 3:** Interindividual baseline variance (CoV_G_), intraindividual variance across time (CoV_T_), reference change value (RCV), and asymmetrical limits of the log-normal RCV (RCV_up_/RCV_down_) for clinical (serum markers MELD, UKELD, APRI, FIB4, ELF); Fibroscan® LSM) and magnetic resonance imaging (MRI) measures

		CoV_G_ (%)	CoV_T_ (%)	RCV (%)	RCV_up_/RCV_down_ (%)
*Clinical measures*					
MELD (stable CC annual)		26.2	8.6 [7.0]	17.6	19.9/-15.6
UKELD (stable CC annual)		6.6	2.2 [1.7]	5.1	5.5/-5.1
APRI (stable CC annual)		104.3	19.4 [10.0]	39.5	51.8/-30.4
FIB4 (stable CC annual)		78.1	17.9 [10.8]	36.4	46.6/-28.6
ELF score	Stable CC annual	10.4	4.0 [3.2]	6.8	8.3/-7.7
	Stable CC b-Y3	8.4	4.2 [3.0]	8.6	9.1/-8.1
	HV b-Y3	7.6	3.6 [2.1]	7.4	7.0/-7.7
*Fibroscan® LSM (Stable CC annual)*		59.8	20.7 [21.6]	48.3	93.0/-41.0
*MRI measures*					
Liver volume	Stable CC annual	26.6	7.5 [3.4]	13.1	16.8/-14.4
	Stable CC b-Y3	21.6	9.4 [8.1]	19.1	21.8/-16.8
	HV b-Y3	9.9	1.8 [5.7]	3.6	3.7/-3.5
Portal vein area	Stable CC annual	31.6	11.0 [9.4]	16.8	22.0/-18.1
	Stable CC b-Y3	22.4	11.0 [9.4]	19.2	21.9/-16.8
	HV b-Y3	22.5	12.4 [8.2]	25.4	21.4/-30.1
Total hepatic blood flow	Stable CC annual	30.2	12.2 [16.8]	26.3	36.9/-26.9
	Stable CC b-Y3	19.9	9.1 [11.5]	18.6	21.1/-16.4
	HV b-Y3	27.8	28.1 [33.4]	65.5	114/-53.4
Liver perfusion	Stable CC annual	31.0	21.0 [14.7]	40.8	61.7/-38.2
	Stable CC b-Y3	16.8	20.6 [22.7]	31.1	42.4/-31.8
	HV b-Y3	29.3	13.1 [20.8]	26.7	32.0/-22.3
Liver T1	Stable CC annual	11.0	4.2 [2.2]	7.3	9.1/-8.4
	Stable CC b-Y3	12.7	5.0 [4.2]	10.2	11.0/-9.6
	HV b-Y3	6.2	2.5 [2.5]	5.1	5.3/-4.9
Spleen volume	Stable CC annual	48.4	9.5 [5.8]	15.3	19.9/-16.6
	Stable CC b-Y3	35.1	11.1 [7.4]	22.6	26.5/-19.4
	HV b-Y3	28.4	14.8 [5.3]	30.1	24.6/-37.0
Splenic artery flow	Stable CC annual	38.6	25.0 [24.5]	46.5	72.9/-42.2
	Stable CC b-Y3	35.2	8.2 [12.5]	16.7	18.7/-14.9
	HV b-Y3	33.4	18.0 [30.2]	36.7	47.1/-28.7
Superior mesenteric artery flow	Stable CC annual	35.3	15.7 [9.7]	31.1	44.5/-30.8
	Stable CC b-Y3	36.8	13.7 [16.9]	28.0	33.9/-23.2
	HV b-Y3	23.1	9.8 [7.9]	20.0	17.4/-22.9
Spleen perfusion	Stable CC annual	31.4	16.0 [9.2]	26.3	36.6/-26.8
	Stable CC b-Y3	36.4	11.4 [21.7]	23.2	27.3/-19.9
	HV b-Y3	27.1	10.4 [3.4]	21.2	18.4/-24.9
Spleen T1	Stable CC annual	7.7	2.8 [3.7]	4.1	5.0/-4.8
	Stable CC b-Y3	5.2	4.2 [3.7]	8.5	9.0/-8.0
	HV b-Y3	5.9	2.3 [2.8]	4.6	4.5/-4.7
Renal cortex T1	Stable CC annual	10.1	3.6 [2.5]	6.6	8.2/-7.6
	Stable CC b-Y3	9.2	3.6 [3.7]	7.4	7.0/-7.7
	HV b-Y3	5.6	2.4 [2.3]	4.9	4.8/-4.7
Cardiac Index	Stable CC annual	26.7	9.5 [7.9]	19.2	25.6/-20.4
	Stable CC b-Y3	27.5	10.9 [10.9]	22.4	26.0/-19.2
	HV b-Y3	20.5	7.4 [9.0]	15.1	16.7/-13.7
LV wall mass index	Stable CC annual	31.8	15.3 [11.2]	33.1	32.4/-47.9
	Stable CC b-Y3	28.3	13.8 [6.9]	28.2	34.1/-23.3
	HV b-Y3	32.6	10.1 [8.9]	20.7	23.8/-18.0

For each parameter, variation indices are given for annual variability in the stable compensated cirrhosis (CC) control group (stable CC annual), and for baseline to year 3 variability in the stable CC control group (Stable CC b-Y3) and the HV group (HV b-Y3)

*CoV* coefficient of variation, *MELD* model for end-stage liver disease, *UKELD* United Kingdom Model for end-stage liver disease, *APRI* aspartate aminotransferase to platelet ratio index, *FIB4* Fibrosis-4, *ELF* enhanced liver fibrosis, *Fibroscan® LSM* Liver stiffness measure, *LV* left ventricle

### Annual intra-individual variability in MRI measures in the stable CC control group

**Fig. 4:**
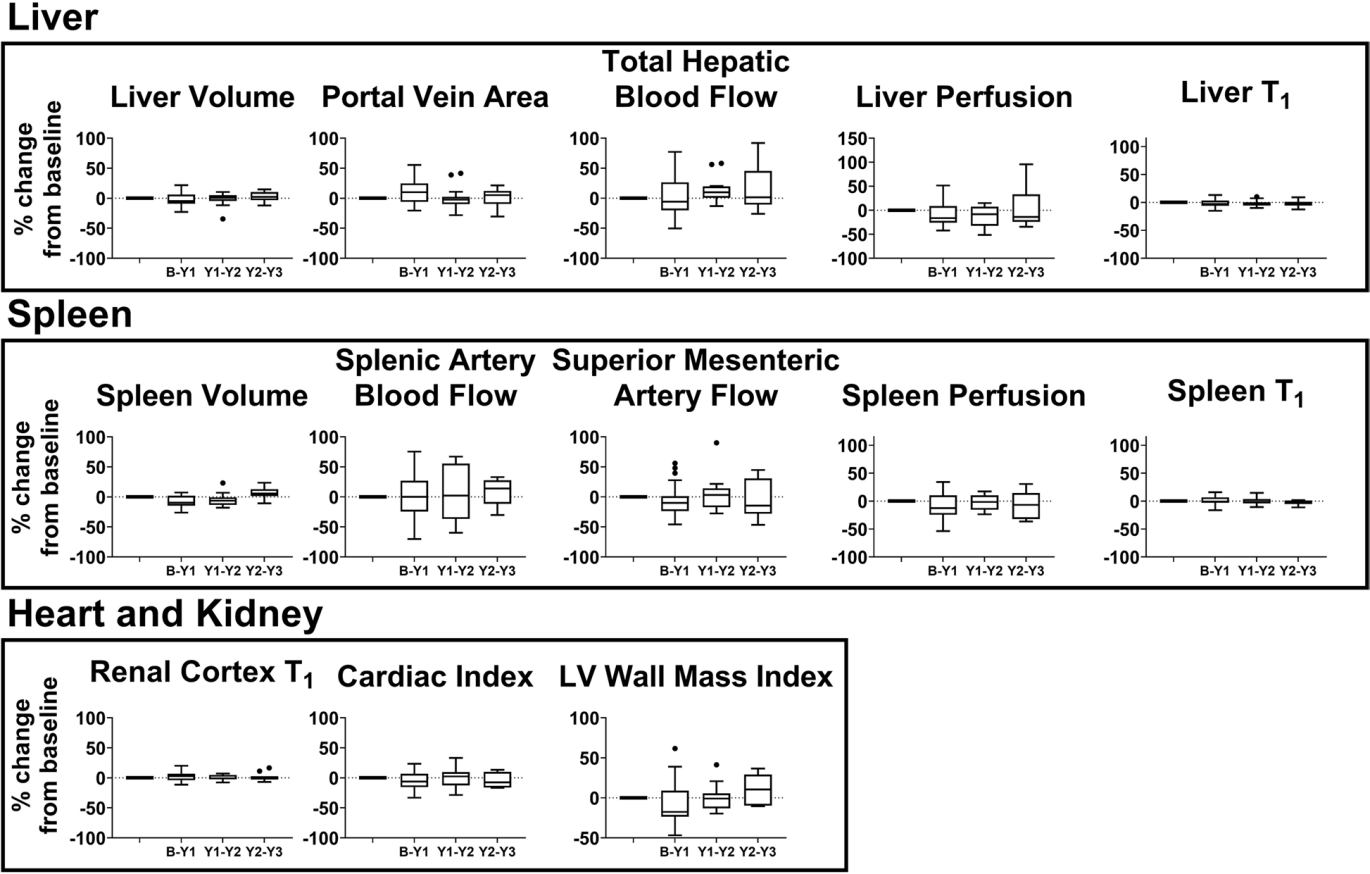
Year-to-year percentage change in magnetic resonance imaging measures in the stable compensated cirrhosis control group. Measures liver (volume, portal vein area, total hepatic blood flow, liver perfusion, liver T1), spleen (volume, splenic and superior mesenteric artery flow, spleen perfusion, spleen T1), kidney (renal cortex T1), and heart (cardiac index and left ventricle [LV] wall mass index) are shown. Bars indicate interquartile range and horizontal bold line shows the median percentage change, dots represent outliers

**Fig. 5:**
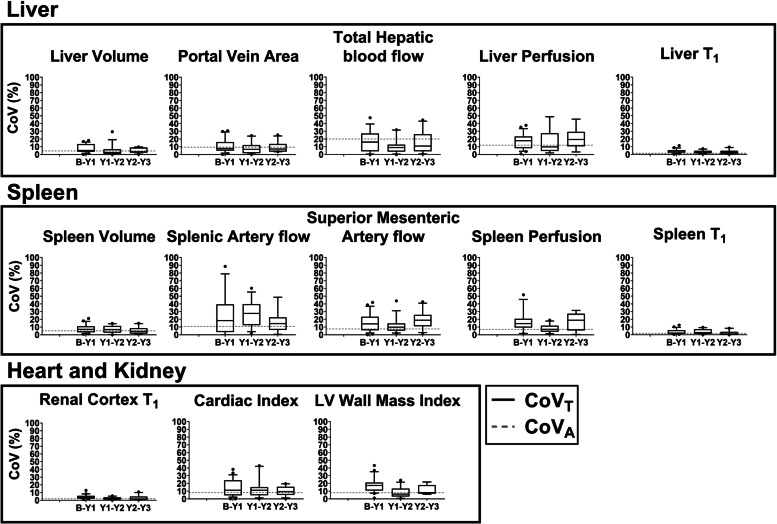
Year-to-year coefficient of variation (CoV_T_) in magnetic resonance imaging measures in the stable compensated cirrhosis control group. Bars indicate the interquartile range and the horizontal bold line shows the median CoV_T_ at each time point, dots represent outliers. The technical variation termed the analytical CoV (CoV_A_) measured in healthy volunteers from triplicate scans collected 1 week apart [10] is shown by the grey dashed line

Figure 4 shows the year-to-year percentage change in MRI measures. In this stable CC control group, there was no significant difference in any MRI measure when compared year-to-year. Figure 5 shows the CoV_T_ of each MRI measure. The annual variation in structural-related MRI measures (volume and T1) is smaller than haemodynamic measures (vessel flow and tissue perfusion). The CoV_T_ of liver and spleen volume was < 10%, whilst the CoV_T_ of liver, spleen and renal cortex T1 was < 5%. Contrastingly, flow measurements of splanchnic circulation (splenic artery and superior mesenteric artery) had a median CoV_T_ of 25% and 16%, respectively. For comparison, in Fig. 5, the technical variation (analytical CoV, CoV_A_) measured in HVs from triplicate scans 1 week apart [10] is shown. Table 3 summarises CoV_G_, CoV_T_, and RCV in annual MRI measures in the stable CC control group. The lowest values of RCV were for liver T1 (7.3%), spleen T1 (4.1%), and renal cortex T1 (6.6%), with the highest RCV values, were seen for liver perfusion (40.8%) and splenic artery flow (46.5%).

**Fig. 6:**
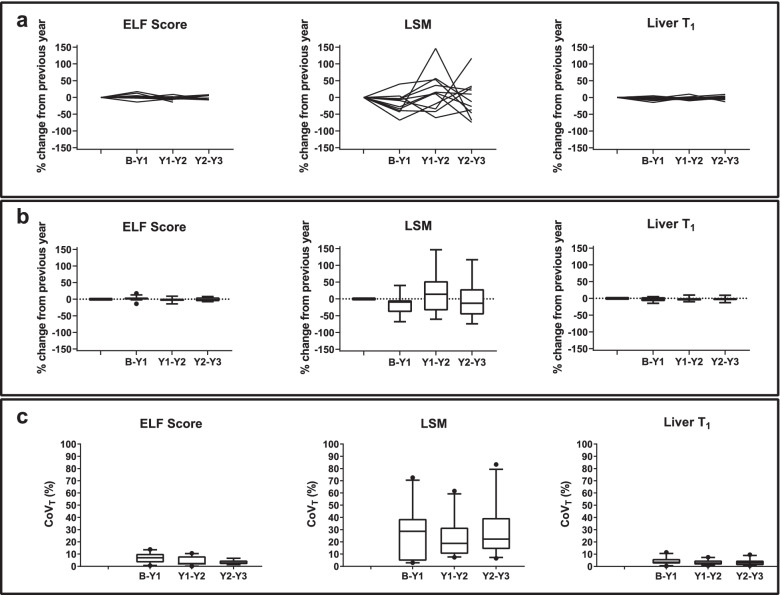
ELF score, Fibroscan® LSM and liver T1 for the stable compensated cirrhosis control patients who completed all three annual follow-up scans: **a** individual subject percentage change from baseline values at year 1, year 2, and year 3; **b** group percentage change from baseline values at year 1, year 2, and year 3. Bars indicate the interquartile range and bold line shows the median percentage change. There was no significant difference from baseline (*p* > 0.05 Bonferroni-corrected); **c** Year-to-year coefficient of variation (CoV_T_) in magnetic resonance imaging measures. Bars indicate the interquartile range and the horizontal bold line shows the median CoV, dots represent outliers. *ELF* Enhanced liver fibrosis, *LSM* Liver stiffness measure

Since Fibroscan® LSM [28], ELF [29], and liver T1 [11] have each been shown to provide a method to evaluate liver fibrosis, Fig. 6 illustrates the year-to-year percentage change and the CoV_T_ in these measures for those stable CC control patients who completed all three annual follow-up scans. All individual ELF score and liver T1 show a percentage change < 17.5% and < 14.6%, respectively, and year-to-year CoV_T_ < 13.8% and < 11.4%, respectively, whilst Fibroscan® LSM had a percentage change of up to 150%, resulting into a CoV_T_ of 83%.

### Baseline to year 3 intraindividual variability in clinical and MRI measures in the stable CC control group and HV group

To assess whether the timing and frequency of sampling are important factors in RCV measures, the CoV_T_ and RCV in clinical and MRI measures for subjects (stable CC and HV) studied between baseline and year 3 were calculated, as shown in Table 3. There was no noticeable difference in RCV values computed for the different frequency of measures (annual *versus* baseline to year 3) for the stable CC control group. RCVs were similar between the stable CC control group and the HV group for baseline to year 3 measures.

### Performance of measures to detect change in disease stage

**Fig. 7:**
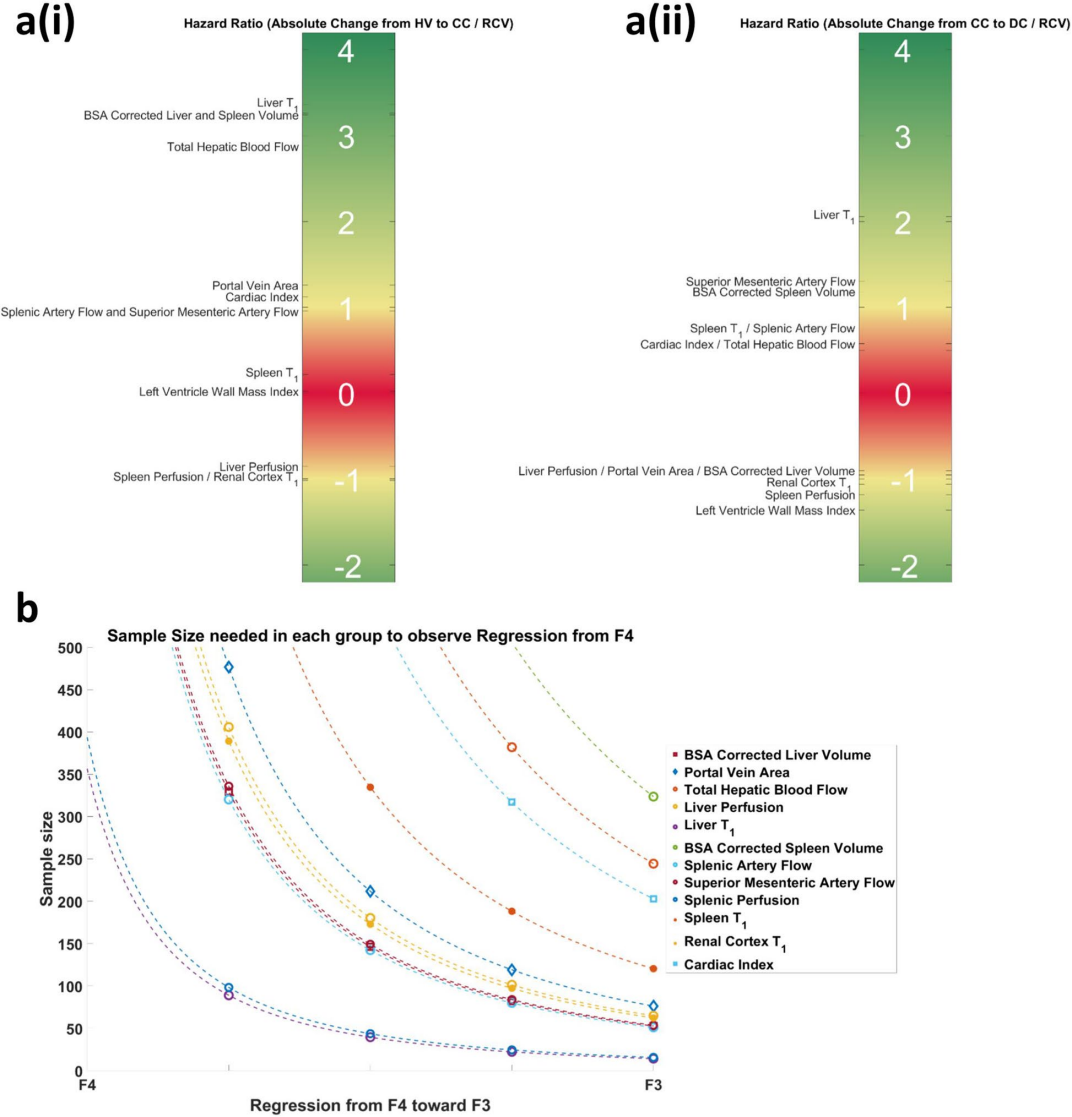
A schematic of the hazard ratio (HR) for disease progression. Panel **a** (i) shows the progression from healthy volunteer (HV) to compensated cirrhosis (CC), whilst panel **a** (ii) shows that the hazard ratio for progression from CC to decompensated cirrhosis (DC). Positive values indicate an increase in measure and negative values a decrease in measures. If the absolute value of the HR is >1 this indicates the reference change value (RCV) is less than the clinical change. Panel **b** shows sample size estimation for the number of CC patients required in clinical trial to detect a change from stage F4 (compensated cirrhosis, CC) to F3 (advanced cirrhosis) liver disease which has clinical significance; data points are shown for a 25%, 50%, 75%, and 100% regression from F4 to F3. *BSA* Body surface area

Figure 7 provides a schematic of the HR for disease progression. Figure 7a (i) shows the progression of HV to CC, for which a HR much higher than 1.0 was found for liver T1, BSA-corrected liver and spleen volume and hepatic blood flow and is ~1.0 for portal vein area, cardiac index, splenic and superior mesenteric artery flow, and ~ -1.0 for liver and spleen perfusion and renal cortex T1. For progression from CC to DC, Fig. 7a(ii) shows that the HR of portal vein area and liver volume changes sign to ~ -1, whilst liver T1 remained much higher than 1.0. Similar HRs were found for other MRI measures between HV to CC and CC to DC, except for LV wall mass which is lower than -1 for CC to DC.

### Estimating sample sizes for clinical trials to predict regression of cirrhosis using multiparametric MRI

Figure 7b shows the sample size of each MRI measure required in a clinical trial to assess cirrhosis regression from F4 toward F3, shown as a percentage change from the mean MRI value in the stable CC group. For a disease state change from F4 to F3, liver T1, spleen and liver volume, and portal volume area require a sample size of lower than 100.

## Discussion

Here we evaluate the serial variation of clinical measures and multi-organ (cardiac, kidney, liver and splanchnic circulation) multiparametric MRI measures that have the potential to study disease-related changes in patients with CC and HVs. Repeatability of quantitative MRI measures has generally been assessed in HVs across vendors and field strength over a period of hours or up to 1 week (*e.g.*, liver T1 and T2*, as reported by Bachtiar et al. [30]). To our knowledge, limited long-term reproducibility data has been collected in only healthy control subjects and serial variation has not previously been evaluated in imaging biomarkers in patients with stable CC. Knowledge of such variability is important since many trials are now beginning to study serial annual MRI changes, for example due to drug treatments [20, 31]. The lack of a control group is often stated as a limitation in such longitudinal trials [31]. In this study, stable CC patients who remained compensated for at least 2 years following their final MRI visit were followed annually to determine the variability in clinical and multiorgan MRI measures.

Among the clinical measures, ELF was most consistent with a year-to-year CoV_T_ < 5% and RCV of 6.8%; in comparison, APRI and FIB4 showed scores with CoV_T_ of 19% and 18%, and RCV of 36% and 39%, respectively. Fibroscan® LSM had a median (IQR) year-to-year CoV_T_ of 20.7% (21.6%) resulting into a large RCV of 40.8%. This level of change agrees with Nascimbeni et al. [19], who reported a retrospective analysis of 500 paired Fibroscan® LSMs with a variation of over 50% in 61 paired measurements. Thus, an increase in LSM of 33% (*i.e.*, from 15 kPa to 20 kPa) would not reflect a true change, which would have implications on clinical management of this patient. A LSM of less than 20 kPa is the threshold set by Baveno VI guidelines [32] to avoid screening gastroscopy for varices, but if LSM subsequently increases on annual follow-up, endoscopy is recommended. In comparison to previous studies, Vergniol et al. [6] showed that the change from baseline to 3 years in LSM from Fibroscan®, APRI and FIB4 has prognostic value in chronic hepatitis. Siddiqui et al. [33] showed that FIB4, APRI, and NAFLD fibrosis scores can detect fibrosis progression, whilst Hartl et al. [34] performed annual LSMs and showed LSM was a reliable predictor in autoimmune hepatitis.

Liver T1 has been shown to provide a marker of liver disease due to increases in extracellular tissue fluid that occurs in response to inflammation and fibrosis, this had a low annual variance of CoV_T_ of 2.5% across the 3-year follow-up period in HVs and 4.2% in CC, with a RCV < 7%. Using LiverMultiScan® in a noncirrhotic population, Harrison et al. [10] showed a similar liver T1 CoV_T_ of 2.3% over an 18-week period. Renal cortex T1, which has been shown to decrease in cirrhosis, and spleen T1 was also consistent over the 3-year period, with a CoVT of 3.6 and 2.8%, and resulting into a RCV < 7%. It should be noted that here we have a fat-suppressed inversion recovery spin-echo echo-planar imaging scheme for T1 mapping rather than a modified Look-Locker inversion recovery, MOLLI, scheme, as the former is not confounded by the effect of iron, fat, and frequency offsets [35]. These measures can be compared to the use of gadolinium ethoxybenzyl-diethylenetriaminepentaacetic acid, Gd-EOB-DTPA, pre- and postcontrast images to assess liver function and the degree of liver fibrosis, where a CoV of 7.1% has been reported in cirrhotic patients [36]. However, it should be also noted that the absence of both previous reaction to MRI contrast media and renal failure is necessary to undergo this contrast-enhanced T1 relaxometry.

The CoV_T_ of haemodynamic measures, which fluctuate with daily physiology, were higher than structural measures, with RCVs of 20–30% for vessel measures and ~50% for organ perfusion. We have previously shown the within-session analytical CoV of phase-contrast MRI measurements of hepatic and splanchnic flow to be 10% [10], and that the RCV includes the biological variation and differences in scan planning. Of note, hepatic and splenic artery flow measurements appear least consistent likely due to the increased difficulty in identifying and planning of these vessels. Here we use a flow alternating inversion recovery-based ASL scheme, which labels both blood from the hepatic artery and portal vein, and accounts for measured T1 in perfusion quantification. The values measured are similar to those reported in dynamic contrast-enhanced studies which show liver perfusion parameters to have a CoV of 39% 1 week apart [37].

The HR plots highlight that those measures which best detect clinical change are liver T1, BSA-corrected liver and spleen volume and total hepatic blood flow for progression from HV to CC, whilst liver T1 and LV wall mass best detected the evolution of CC into DC. Portal vein area and liver volume MRI measures increased from a healthy state to CC, and then decreased as DC occurs, so particular care should be taken when assessing these measures as a reduction in a marker could simultaneously indicate progression or regression. However, these markers used in combination with markers that progress/regress linearly could help provide better individual patient care.

New drugs are now becoming available to study CC regression/nonprogression [38], and here we showed those MRI measures suited to monitor such changes in clinical trials, with liver T1, spleen and liver volume and portal volume being the best candidates.

The main limitations of our study relate to the relatively low sample size and the dropout of patients through the follow-up period. As with all longitudinal studies, we encountered patient attrition which resulted in fewer patients at the end of the study. However, this is the first prospective study to attempt to specifically address the question of the biological variation in noninvasive markers in cirrhosis.

In conclusion, we provided the CoV_G_, CoV_T_, and RCV for clinical and MRI measures in stable CC patients. This is the first time that detailed serial noninvasive MRI measures have been reported. The RCVs can be used to interpret the change in measures in CC patients. We have used these to estimate sample size to power future clinical trials of cirrhosis regression using multiparametric MRI measures.

## Data Availability

The data supporting the findings of this research can be requested by approaching the corresponding author.

## Abbreviations

APRI: Aspartate aminotransferase to platelet ratio index
ASL: Arterial spin labelling
BMI: Body mass index
BSA: Body surface area
CC: Compensated cirrhosis
CoV: Coefficient of variation
CoV_A_: Analytical coefficient of variation
CoV_G_: Groupwise coefficient of variation
CoV_T_: Coefficient of Variation across time
DC: Decompensated cirrhosis
ELF: Enhanced liver fibrosis
FIB4: Fibrosis-4
HR: Hazard ratio
HV: Healthy volunteer
IQR: Interquartile range
LSM: Liver stiffness measure
LV: Left ventricle
MELD: Model for end stage liver disease
MRI: Magnetic resonance imaging
mpMRI: Multiparametric MRI
NAFLD: Non-alcoholic fatty liver disease
RCV: Reference change value
UKELD: United Kingdom model for end-stage liver disease
